# Trajectory of severe COVID anxiety and predictors for recovery in an 18-month UK cohort

**DOI:** 10.1192/bjo.2025.10884

**Published:** 2025-11-03

**Authors:** Jacob D. King, Aisling McQuaid, Kirsten Barnicot, Paul Bassett, Verity C. Leeson, Martina Di Simplicio, Peter Tyrer, Helen Tyrer, Richard G. Watt, Mike J. Crawford

**Affiliations:** Division of Psychiatry, Imperial College London, UK; School of Health Sciences, City University of London, UK; StatsConsultancy Ltd, Amersham, UK; Epidemiology and Public Health, University College London, UK

**Keywords:** Anxiety disorders, health behaviour, health anxiety, pandemic

## Abstract

**Background:**

People with severe COVID anxiety have significant fears of contagion, physiological symptoms of anxiety in response to a COVID stimulus and employ often disproportionate safety behaviours at the expense of other life priorities.

**Aims:**

To characterise the long-term trajectory of severe COVID anxiety, and the factors that influence recovery.

**Method:**

This prospective cohort study followed 285 people with severe COVID anxiety in the UK over 18 months. A nested randomised feasibility trial tested an online cognitive–behavioural therapy (CBT)-based intervention (no. ISRCTN14973494). Descriptive statistics and linear regression models identified factors associated with change in COVID anxiety over 18 months.

**Results:**

Most participants experienced major reductions in COVID anxiety over time (69.8% relative cohort mean decrease, *P* < 0.001), but a quarter of people (23.7%, 95% CI: 17.8–30.1) continued to worry about COVID every day, and for 13% symptoms remained severe even after the ending of all public health restrictions. Increasing age, being from a minority ethnic background that confers greater risk from COVID-19, and the persistence of high levels of health anxiety and depressive symptoms, predicted slower improvements in severe COVID anxiety after adjusting for other clinical and demographic factors. Neither a trial CBT-based intervention, nor contextual factors including daily case rates, vaccination status or having contracted COVID-19, appeared to affect the trajectory of severe COVID anxiety.

**Conclusions:**

For most people severe COVID anxiety improves significantly with time. However, interventions treating depression and health anxiety, and targeting older people and those from greater-risk minority backgrounds, warrant further investigation in future pandemics.

Anxious thoughts and feelings about COVID-19 are common, and appropriate given the very real threats to health and well-being posed by the virus. Nonetheless, for around 5–15% of people at the height of the pandemic, fears of contagion and anticipation of adverse physical and socioeconomic consequences became overwhelming, leading to changes in behaviour that were no longer adaptive, and reinforced preoccupation with contagion.^1,2^ These included excessive hand-washing, disinfecting items brought into the home, great consumption of COVID-related news media, online symptom checking and not leaving the home to a degree disproportionate to public health guidance, which stopped people from being able to get on with other life priorities.^3,4^ Moreover, somatic symptoms of anxiety such as shortness of breath and gastrointestinal disturbance were sometimes misinterpreted as signs of COVID-19 itself, further reinforcing bodily vigilance and symptom-checking behaviours that perpetuated anxiety.^5,6^

Greater levels of COVID anxiety are associated with poorer levels of overall mental health, socio-occupational functioning and a lower quality of life, as well a greater rates of health anxiety, post-traumatic stress, obsessive–compulsive and depressive symptoms^3,7^ and substance misuse.^8^ In previous work among people with severe levels of COVID anxiety, high levels of co-occurring psychopathology were the norm, and few people scored low on assessment tools measuring depressive, obsessive–compulsive symptoms or generalised anxiety and health anxiety.^3,9^

Put simply, COVID anxiety is best formulated as a specific anxiety syndrome response to COVID-19 stimuli.^10^ It appears to be a heterogenous experience, insofar as there have emerged different underlying psychological processes at play, including variable stimulus hypervigilance and avoidance responses, worrying and checking cycles, differential conditioning and fear generalisation, and post-traumatic-like responses. Indeed COVID anxiety has been formulated variably by different research groups, and probably represents a variety of anxiety processes different for different people: a specific phobia of the coronavirus, a stimulus within generalised or health anxiety or obsessive–compulsive processes, as the trigger for post-traumatic phenomena, or indeed a social stressor that some people with low coping skills, intolerance of uncertainty and high-trait neuroticism are unable to manage. Moreover, specific clinical clusters of severe COVID anxiety have emerged, associated with patterns of associated health behaviours, health conditions that increase risk from COVID-19, symptoms of other mental health disorders and demographic profiles.^11^ Nevertheless, whatever the clinical formulation, given the levels of distress and dysfunction, there is a case to be made that people with severe COVID anxiety might benefit from targeted mental health support.

There has now been much written on the changes in population general mental health, before, during and following the coronavirus pandemic, with near global consistence in patterns of clinically significant increases in population levels of psychological distress and specific anxiety disorders which, having peaked in the early stages of the pandemic, followed a gradual return to near pre-pandemic levels.^12^ However, specific groups of people appear to experience much slower improvements in their mental health, most notably adolescents and young people,^13^ and also individuals who had existing poor mental health states prior to the pandemic.^14^ Given that COVID anxiety is a response to a specific stimulus, it is unclear whether the trajectory of this phenomenon would follow a similar path over the pandemic as case numbers rose and fell as, for example, measures of depression or generalised anxiety do, or indeed whether similar factors influence the trajectory.

A handful of longitudinal studies reporting the level of COVID anxiety (and comparable concepts including coronaphobia, COVID stress disorder/syndrome, COVID-19 fear, COVID-related worries or COVID-19 anxiety syndrome) in the same people over time have been published.^15–18^ However, none have examined which factors are predictive of a change in symptoms, nor do these studies consider specifically people with the most severe and clinically important levels of COVID anxiety. These studies ranged in their follow-up periods from between 5 and 16 months and cover a time period from February 2020 until October 2021 in Brazil, China, the Netherlands, UK and USA. Three studies assessed symptoms at more than two time points. Although all studies demonstrated meaningful reductions in the level of COVID anxiety over time, there do not appear to be studies that have assessed levels of COVID anxiety at a time after the coronavirus became endemic, when there were no longer public health restrictions in place and cases were not routinely monitored. Moreover, since the latest follow-up time point of these longitudinal studies there has been much greater uptake of vaccination, the development and availability of effective treatments and declining mortality rates, all factors likely to be associated with reducing levels of COVID anxiety.

In response to COVID anxiety, there have been a handful of interventions tested, most commonly based on cognitive–behavioural therapy (CBT),^19–21^ each showing some benefit to measures of COVID anxiety against control conditions but with very short follow-up periods – usually a matter of a few weeks. Assessing such interventions with longer-term follow-up may help inform the design and implementation of targeted pandemic mental health care in the future.

In short, the natural course of severe COVID anxiety over longer follow-up periods is unclear, as are the factors that might be associated with improvement or persistence of these symptoms over time and post-pandemic. In this study we therefore report on 18-month outcome data from a cohort of people sampled from the general UK population with severe levels of COVID anxiety, covering a period when COVID had become endemic, and examine the effect of a range of demographic and clinical factors, as well as a small trial intervention, on its trajectory.

## Method

The ‘COVID Anxiety Project’ is an 18-month prospective cohort study, with a nested randomised feasibility trial of an online CBT-based intervention targeting health anxiety for a small subsample.

The project recruited adults from the UK with severe COVID anxiety during the British second and third waves (January–September 2021). This was a time more than a year after the first UK cases were identified, during which the third national lockdown had ended and social distancing restrictions were eased, vaccination was increasingly available but case rates remained high. This time period was selected in order to identify people for whom COVID anxiety symptoms were most likely to be persistent and not largely attributable to an initial, adaptive, ‘uncertainty effect’. For most of the population, uncertainty about the potential social and health impacts in the early pandemic stages was understandably worrying, but most people were able to adapt after some months, seeing major improvements in distress when the risks and solutions became more clear.^12^

Participants were followed up 3, 6 and 18 months after recruitment, with the last follow-up completed in April 2023 when there was no longer national tracking of case rates and no public health restrictions in place.

Analyses of 6-month outcome data from this cohort have previously been published, which demonstrated meaningful declines in the level of COVID anxiety of participants, albeit most continued to experience clinically important levels.^9^ While there were high rates of co-occurring mental health symptoms in this sample, there were notable decreases in almost all psychopathology measures. People who continued to report high levels of health anxiety and depressive symptoms were less likely to have experienced meaningful reductions in their levels of COVID anxiety in multivariate models. Participants received a £10 e-voucher for completion of the baseline survey and a £20 e-voucher for completion of the surveys at 6- and 18-month follow-ups.

### Participants

A total of 1068 individuals who self-identified as being anxious about COVID initially responded to advertisements on social media platforms, and from text message contact made by 19 affiliated general practice (family medicine) clinics in London.^22^

Of that total, 306 people (30.4%) scored 9 or more on the 20-point COVID Anxiety Scale (CAS)^23^ indicating severe COVID anxiety, provided written consent and met other eligibility criteria, including not having a history of psychotic illness nor having had COVID-19 infection in the preceding 4 weeks.^22^ People who additionally scored >20 on the short Health Anxiety Inventory (indicating clinically significant health anxiety symptoms), and who were not currently receiving psychological treatment, were invited to participate in a nested randomised feasibility trial of CBT for Health Anxiety (CBT-HA). The cohort recruited participants until there were sufficient numbers meeting these further inclusion criteria to power the nested randomised feasibility trial.^9^ Randomisation of 40 participants at a ratio of 1:1 would be sufficient to detect a 50% uptake of CBT-HA with 95% confidence intervals of ±15%.

**Fig. 1:**
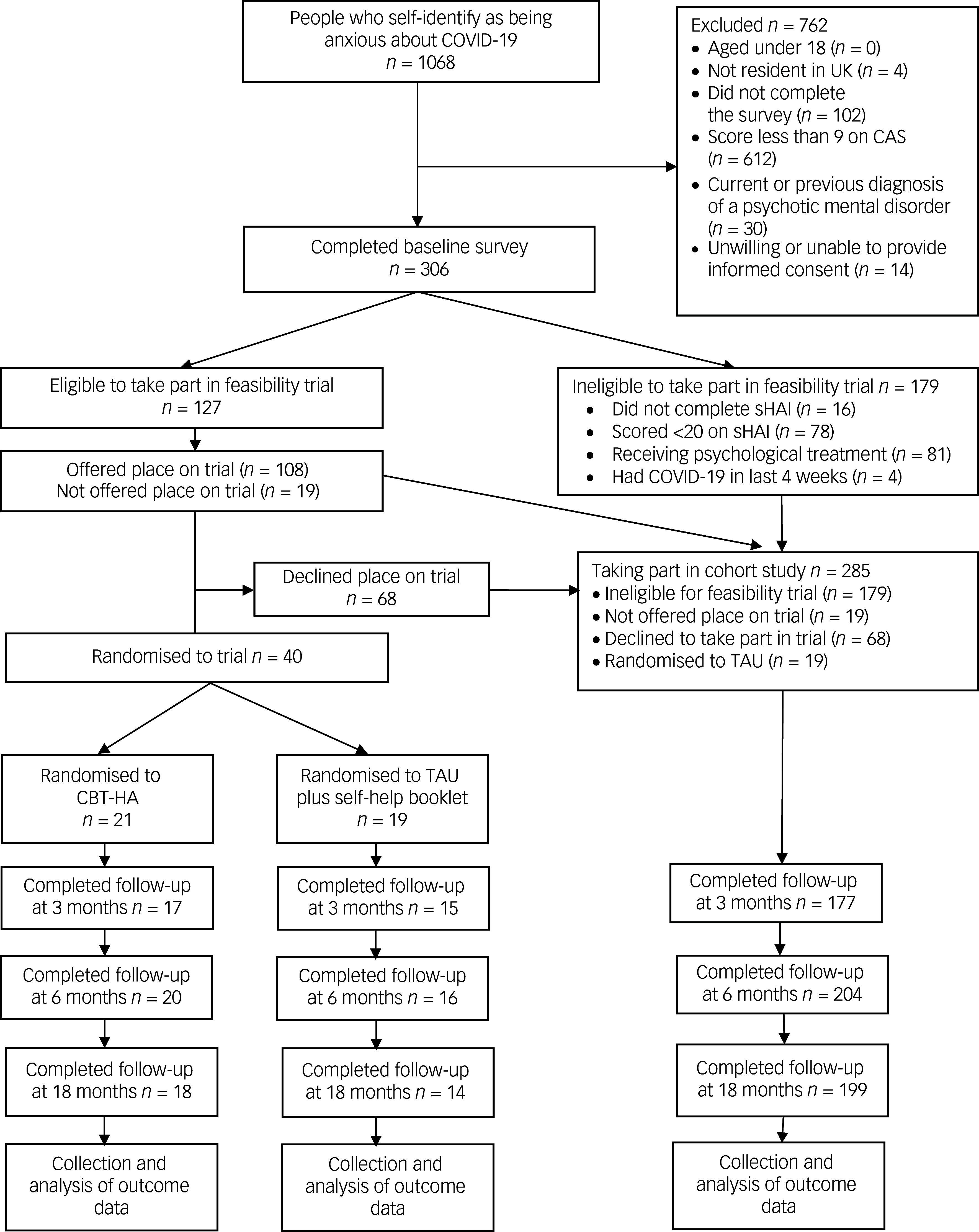
Flow diagram of trial procedure. CAS, COVID Anxiety Scale; CBT-HA, cognitive–behavioural therapy for health anxiety; sHAI, Short Health Anxiety Inventory; TAU, treatment as usual.

This sample was predominantly female (81.2%), with a median average age of 41 years (interquartile range (IQR) 28–53, range 18–83). Compared with the UK general population there was a similar median age, although the age distribution of this sample of people with severe COVID anxiety was positively skewed: while 20% of the sample was aged between 18 and 25 years, 13.9% was over 60. Of those reporting their ethnicity, 29.8% came from an ethnic minority group, about half of whom were of an ethnic group with greater risk to heath from COVID-19. This included people from a Black African, Black Caribbean and South Asian background. There were significant rates of mental health conditions in this cohort, and otherwise nationally representative rates of at-risk health conditions. A full description of baseline cohort characteristics has been reported previously.^3^ Latent profile analysis conducted on baseline data from this cohort identified four clinically distinct clusters of people with severe COVID anxiety.^11^ Clusters with significantly lower and higher burdens of co-occurring mental health symptoms emerged, along with a cluster of individuals with greater than expected rates of at-risk health conditions who were older than cohort average and abstinent of alcohol, and a fourth with prominent obsessive–compulsive symptoms and personality pathology described as ‘anankastic’ (Fig. 1).

### Measures

Our primary outcome was the level of COVID anxiety as measured using the self-report 5-item COVID Anxiety Scale, which was the first, and at the time only, validated assessment scale. This scale assesses the frequency of five somatic symptoms (loss of appetite, gastrointestinal disturbance, poor sleep, dizziness and feeling paralysed or frozen) experienced in response to COVID,^24^ such as watching or reading news about the pandemic. A score of 5 or more indicates the probable presence of levels of COVID anxiety associated with significant disruption to daily life, and a score of 9 or more indicates a severe degree of symptomatology.^25^ At baseline, self-completed questionnaires were used to assess for symptoms of generalised anxiety, depression, health anxiety, obsessive–compulsive symptomatology, personality disorder and drug and alcohol use. Respectively, these were the Generalised Anxiety Disorder scale – 7 items (GAD-7), the Patient Health Questionnaire – 9 items (PHQ-9), the 14 health anxiety items of the Short form Health Anxiety Inventory (sHAI), the Obsessive–Compulsive Inventory – Revised (OCI-R), the Standardised Assessment of Personality Abbreviated Scale (SAPAS), drug use with the Single Drug Use Item (SDUI) and the Alcohol Use Disorders Identification Test for Consumption (AUDIT-C). At baseline none of the assessment scales were meaningfully correlated in this cohort (*r* > 0.6), and other characteristics of these data are presented in the baseline paper.^3^

Additionally we collected a measure of social and occupational functioning (Work and Social Adjustment Scale, WSAS), as well as novel measures of COVID-specific health behaviours including hand-washing, staying at home, disinfecting items brought in to the home and consuming media relating to COVID-19, which were developed in association with people with lived experience of poor mental health during the pandemic.^3^ Questions assessing these behaviours were constructed on 4-point Likert-item scales ranging from 0 (behaviour not used; for example, washing hands to the same degree as before the pandemic) to 4 (dysfunctional form of the behaviour employed; for example, constantly washing one’s hands). In this study we report COVID-associated behaviours in two ways: as the proportion of people employing the dysfunctional form of the behaviour, and also as a mean cohort average score.

Demographic factors were collected including age, gender, ethnicity, employment and relationship status and household composition, as well as self-disclosed physical health conditions, which were coded using MedDRA criteria (https://www.meddra.org/) by a physician. Health conditions and participants’ ethnic background were assessed as increasing an individual’s risk to COVID-19 with the QCOVID tool (https://qcovid.org/). Twenty-one participants were excluded from these cohort analyses because they were randomised to the active arm of the feasibility trial for CBT-HA, which we anticipated would influence the natural trajectory of severe COVID anxiety, and were therefore analysed separately.^22^

At the 18-month follow-up we collected repeat measures for COVID anxiety, generalised and health anxiety, depression, drug and alcohol use and social-occupational dysfunction. All data were collected using the Qualtrics platform (Qualtrics LLC, Seattle, WA, USA; https://www.qualtrics.com/en-gb/).

Serious adverse events (SAEs), which included death, hospitalisation and life-threatening events, were collected at baseline and each of the follow-up periods. Therapists were also reminded to report SAEs that occurred during treatment.

### Randomisation and trial methods

Forty participants who additionally scored over 20 on the HAI (indicating clinically significant health anxiety symptoms), and who were not already engaging in a psychological intervention, were randomised 1:1 in the feasibility trial by the ‘sealed envelope’ method. Both of these arms could continue to access treatment as usual (TAU), including having access to mental health support from primary or secondary care services. Participants were also given a short information guide on maintaining good mental health during the pandemic, developed by psychologists from Central and North West London NHS Foundation Trust. The trial was registered prospectively (https://www.isrctn.com/ISRCTN14973494). Twenty-one participants were randomised to the active arm, and 19 to TAU.

### Intervention

Those 21 individuals in the active arm were offered up to 10 sessions of CBT targeting COVID health anxiety based on a published treatment manual.^26^ This was delivered remotely using a secure web-based telecoms platform. Session content included collaborative CBT formulation about COVID anxiety-specific thoughts and behaviours, symptom diary keeping, techniques to manage distress and reduce reinforcing behaviours and graded exposure techniques, among others. All therapists had a degree in a health-related subject, as well as past experience of delivering psychological interventions; they received a 90 min training session in CBT-HA from H.T., an expert in the treatment of health anxiety, and were required to attend fortnightly clinical supervision with her. A full description of the intervention has been reported in the trial protocol.^22^ Active arm participants received all assigned intervention sessions before the 6-month follow-up mark.

### Data analysis

We published a statistical analysis plan in the ISRCTN registry prior to analysis (no. ISRCTN14973494). There were no within-scale missing data because the data collection platform that we used required that participants complete all items before they could progress through the survey.

We used descriptive statistics and linear regression models to assess the change in CAS scores from baseline to the 18-month follow-up. Univariate analysis first examined the unadjusted associations between change in CAS score and demographic factors, and also clinical factors both as baseline predictors and reductions in scores in the clinical measures between baseline and the 18-month time point. We used a backwards selection procedure in multivariate analysis, looking at the joint association of only those factors found to be associated with the outcome in univariate analysis (*P* < 0.2). The scores of feasibility trial participants were reported descriptively, and paired *t*-tests were used to assess the significance between baseline scores and the 18-month follow-up time point.

We also conducted *ad hoc* off-protocol analyses to examine the association of national daily COVID-19 cases and COVID-19-associated hospitalisations with the severity of COVID anxiety in our cohort during the study period. Seven-day rolling averages of the number of COVID-19 cases reported, and COVID-19-associated hospital admissions in England and Wales (representing 90% of UK population), were taken from publicly available UK government data (https://ukhsa-dashboard.data.gov.uk/). Scotland and Northern Ireland had stopped daily reporting of figures in May 2022. We aligned, by date, 865 unpaired CAS data points from our study to the 7-day rolling average COVID-19 cases and hospitalisations between January 2021 and April 2023. We used univariate analyses to assess the unadjusted association between these rates and CAS scores, and multivariate analysis to assess the mediating effect of other factors.

All data were managed and analysed in SPSS version 20.0 (SPSS Inc., Chicago, IL, USA; https://www.ibm.com/products/spss) and Stata version 16.1 (StataCorp LLC, College Station, TX, USA; https://www.stata.com/products/).

### Ethical approval

This study was approved by the Leicester Central Research Ethics Committee and Health Regulation Authority in 2020 (reference no. 20/EM/023). The authors assert that all procedures contributing to this work comply with the ethical standards of the relevant national and institutional committees on human experimentation, and with the Helsinki Declaration of 1975 as revised in 2013. Following recruitment, all individuals were required to read and sign a consent form before participation, and received a copy.

## Results

Of 285 participants, 177 (61.2%) provided responses at 3-month follow-up, 204 (71.2%) at 6 months and 199 (69.8%) at 18 months. Men (*P* = 0.008), people of a Black, South Asian or mixed ethnic heritage (*P* = 0.03), people with baseline scores indicating lower levels of personality pathology (*P* = 0.03) and those in the subgroup without high rates of co-occurring psychopathology (*P* = 0.03) were less likely to have completed follow-up at 18 months.

Cohort mean average CAS scores dropped from 12.4 (95% CI: 12.1−12.7) to 7.7 (95% CI: 7.1−8.4) at 3 months, to 6.8 (95% CI: 6.2−7.4) at 6 months and to 3.7 (95% CI: 3.2−4.2) at 18 months (69.8% relative decrease, *P* < 0.001); 13.1% (*n* = 26) of the cohort continued to score over 9 (indicating severe COVID anxiety) at 18 months, and 38.2% (*n* = 76) continued to score 5 or more indicating levels of COVID anxiety associated with an impact on daily life. Sixty-one (31%) people scored 0 at 18 months, indicating resolution of COVID anxiety symptoms.

The cohort demonstrated major reductions in mean generalised anxiety (−31.7%, *P* < 0.001), depressive symptoms (−24.5%, *P* < 0.001), health anxiety (−23.9%, *P* < 0.001) and social and occupational dysfunction (−43.0%, *P* < 0.001) over the 18 months (Table 1).

**Table 1:** Changes in key parameters over time

Variable	Time point	*n*	Mean ± s.d.	Change, mean (95% CI)	*P*-value versus baseline
CAS	Baseline	285	12.4 ± 3.0	0	
	3 months	177	7.7 ± 4.4	−4.6 (−5.2, −4.1)	<0.001
	6 months	204	6.8 ± 4.5	−5.5 (−6.0, −4.9)	<0.001
	18 months	199	3.7 ± 3.9	−8.7 (−9.2, −8.1)	<0.001
WSAS	Baseline	267	21.3 ± 7.7	0	
	3 months	169	19.0 ± 9.0	−2.7 (−3.9, −1.4)	<0.001
	6 months	203	18.3 ± 9.0	−3.3 (−4.5, −2.1)	<0.001
	18 months	199	12.1 ± 9.5	−9.4 (−10.6, −8.2)	<0.001
GAD-7	Baseline	274	15.4 ± 4.0	0	
	3 months	172	13.4 ± 5.0	−2.1 (−2.9, −1.4)	<0.001
	6 months	203	12.5 ± 5.5	−3.0 (−3.7, −2.3)	<0.001
	18 months	199	10.5 ± 6.1	−5.1 (−5.8, −4.4)	<0.001
sHAI	Baseline	269	23.2 ± 7.1	0	
	3 months	171	21.7 ± 8.5	−1.8 (−2.6, −0.9)	<0.001
	6 months	203	19.7 ± 8.4	−3.5 (−4.3, −2.6)	<0.001
	18 months	199	18.2 ± 8.1	−5.0 (−5.8, −4.1)	<0.001
PHQ-9	Baseline	268	15.6 ± 5.5	0	
	3 months	170	13.8 ± 6.4	−1.5 (−2.3, −0.6)	0.001
	6 months	203	13.4 ± 6.7	−2.1 (−2.9, −1.3)	<0.001
	18 months	199	11.7 ± 7.2	−4.0 (−4.8, −3.1)	<0.001
OCI-R	Baseline	264	30.4 ± 15.3	0	
	3 months	169	28.2 ± 16.8	−1.5 (−3.2, 0.2)	0.09
	6 months	203	25.9 ± 15.5	−4.1 (−5.7, −2.5)	<0.001
	18 months	^a^			
AUDIT-C	Baseline	264	2.66 ± 2.97	0	
	3 months	173	2.48 ± 2.77	0.05 (−0.21, 0.31)	0.70
	6 months	204	2.41 ± 2.62	0.03 (−0.21, 0.27)	0.82
	18 months	199	2.27 ± 2.68	−0.20 (−0.44, 0.04)	0.10

Variable	Time point	*n*	Mean score	Mean % change (from baseline)	*P*-value versus baseline
Quality of life (EQ-5D-3L)	Baseline	267	0.498	–	–
	3 months	167	0.527	+5.88	NA
	6 months	203	0.532	+6.89	NA
	18 months	a			

Variable	Time point	*n*	*n* (%)	Odds ratio (95% CI)	*P*-value versus baseline
Drug use	Baseline	264	24 (9.1)	1	
	3 months	173	12 (6.9)	0.63 (0.20, 1.95)	0.43
	6 months	204	20 (9.8)	1.59 (0.59, 4.24)	0.36
	18 months	199	15 (7.5)	0.77 (0.27, 2.13)	0.61

CAS, COVID Anxiety Scale; GAD-7, General Anxiety Disorder Assessment 7-item; PHQ-9, Patient Health Questionnaire 9-item; OCI-R, Obsessive–Compulsive Inventory Revised; sHAI, Health Anxiety Inventory Short Form; AUDIT-C, Alcohol Use Disorders Identification Test of Consumption; WSAS, Work and Social Adjustment Scale; EQ-5D-3L, EuroQol 5-Dimension 5-Level measure of health-related quality of life; NA, not applicable.

a . Data not collected.

At the 18-month follow-up, almost half of the sample reported still washing their hands much more than before the start of the pandemic (47.9%, 95% CI: 40.6−55.3%), 1 in 5 continued to disinfect most items coming into their home (21.6%, 95% CI: 16.0−28.1%), another 1 in 5 reported watching COVID-related news media at least daily (22.1%, 95% CI: 16.4−28.7%) and almost a quarter (23.7%, 95% CI: 17.8−30.1) reported that they still worried about COVID every day. Of those participants with school-age children, 11.2% (*n* = 7) continued not to send their children to school even though they were expected to, because of concerns about COVID contagion. By the 18-month follow-up, 124 (62.9%) participants reported having been infected by the coronavirus and 9 (4.6%) reported having been hospitalised by it; 175 (88.8%) reported receiving at least one COVID vaccine (Table 2).

**Table 2:** Changes in COVID-related thoughts and behaviours over time

COVID-related behaviour	Time point	*n*	*n* (%)	Odds ratio (95% CI)	*P*-value versus baseline
Constant COVID worry	Baseline	268	57 (21.3)	1	–
	3 months	168	24 (14.3)	0.62 (0.37–1.04)	0.07
	6 months	203	26 (12.8)	0.54 (0.33–0.90)	0.02
	18 months	198	5 (2.5)	0.10 (0.034–0.24)	<0.001
Constantly watching COVID news	Baseline	268	31 (11.6)	1	–
	3 months	168	11 (6.5)	0.54 (0.26–1.1)	0.09
	6 months	203	9 (4.4)	0.35 (0.16–0.76)	0.008
	18 months	198	4 (2.0)	0.16 (0.054–0.45)	<0.001
Never leaving home	Baseline	268	31 (11.6)	1	–
	3 months	168	14 (8.3)	0.70 (0.36–1.35)	0.28
	6 months	203	19 (9.4)	0.79 (0.43–1.44)	0.44
	18 months	198	10 (5.1)	0.40 (0.19–0.85)	0.02
Not sending children to school^a^	Baseline	94	19 (20.2)	1	–
	3 months	53	10 (18.9)	0.92 (0.39–2.15)	0.84
	6 months	66	7 (10.6)	0.46 (0.19–1.19)	0.11
	18 months	61	7 (11.5)	0.51 (0.20–1.30)	0.16
Buying all food online	Baseline	268	100 (37.3)	1	–
	3 months	168	61 (36.3)	0.96 (0.64–1.43)	0.83
	6 months	203	53 (26.1)	0.59 (0.4–0.88)	0.01
	18 months	198	39 (19.7)	0.41 (0.27–0.63)	<0.001
Constantly washing hands	Baseline	268	54 (20.1)	1	–
	3 months	168	41 (24.4)	1.28 (0.81–2.03)	0.30
	6 months	203	31 (15.3)	0.71 (0.44–1.16)	0.17
	18 months	198	23 (11.6)	0.52 (0.31–0.88)	0.02
Washing all items coming into the home	Baseline	268	83 (31.0)	1	–
	3 months	168	39 (23.3)	0.67 (0.43–1.04)	0.08
	6 months	203	37 (18.2)	0.50 (0.34–0.77)	0.002
	18 months	198	24 (12.1)	0.31 (0.19–0.51)	<0.001
Washing each item of clothing when worn outside	Baseline	268	47 (17.5)	1	–
	3 months	168	29 (17.3)	0.98 (0.59–1.63)	0.94
	6 months	203	27 (13.3)	0.72 (0.43–1.20)	0.21
	18 months	198	21 (10.6)	0.56 (0.32–0.97)	0.04

a . Of those respondents with school-aged children.

When adjusted for baseline CAS score, increasing age was the only demographic factor that remained significant (*P* = 0.005), at an alpha level of 0.05 in univariate analysis, as being predictive of slower improvements in COVID anxiety symptoms over time. While baseline levels of generalised anxiety, depression, personality pathology, obsessive–compulsive symptoms and alcohol use were also not predictive of a differing trajectory, improvements in generalised anxiety, health anxiety and depression over time were favourably and significantly associated with a CAS reduction. Other variables, including living with an at-risk health condition or living with someone vulnerable to COVID-19, were not associated with smaller reductions in COVID anxiety over time. There was no predictive effect of subgroup cluster on the magnitude of CAS reduction, nor was there an effect of vaccination status or having contracted COVID-19.

In multivariate modelling, age and coming from a Black, South Asian or mixed ethnic background were significant predictors of lesser reductions in CAS, compared with people from ethic backgrounds without an increased risk (White British/other and East Asian). Every 10-year increment increase in age was associated with a 0.4-point smaller reduction in CAS score over 18 months. Furthermore, individuals for whom depressive and health anxiety symptoms reduced more over the follow-up period also had significantly greater reductions in their COVID anxiety (Table 3).

**Table 3:** Associations between demographic and clinical factors, and reduction in CAS scores from baseline to 18-month follow-up

Factor/baseline score	Category	CAS reduction	Univariable analysis		Multivariable analysis	
		Mean ± s.d.	Coeff. (95% CI)	*P*-value	Coeff. (95% CI)	*P*-value
Age (*)	–	–	−0.5 (−0.9, −0.2)	0.005	−0.4 (−0.7, −0.1)	0.02
Gender	Female	8.4 ± 4.4	0	0.52	–	–
	Male	9.7 ± 4.3	−0.5 (−2.1, 1.0)			
Ethnicity	Not at greater risk	8.6 ± 4.3	0	0.06	0	0.002
	At greater risk	9.0 ± 4.8	−1.5 (−3.1, 0.9)		−2.2 (−3.6, −0.8)	
Employment	Not employed	8.4 ± 4.1	0	0.29	–	–
	Employed	9.0 ± 4.6	0.6 (−0.5, 1.7)			
Lives alone	No	8.8 ± 4.3	0	0.07	0	0.08
	Yes	7.8 ± 4.8	−1.4 (−2.9, 0.1)		−1.2 (−3.6, −0.8)	
Lives with vulnerable person	No	8.6 ± 4.5	0	0.23	–	–
	Yes	8.8 ± 4.2	−0.7 (−1.8, 0.4)			
At-risk condition	No	8.7 ± 4.4	0	0.69	–	–
	Yes	8.7 ± 4.1	−0.3 (−1.5, 1.0)			
Patient classification^a^	Cluster 1, simple	8.0 ± 4.3	0	0.34	–	–
	Cluster 2, at-risk	8.8 ± 3.7	0.1 (−1.3, 1.4)			
	Cluster 3, anankastic	8.5 ± 5.1	−0.8 (−2.7, 1.0)			
	Cluster 4, complex	10.1 ± 4.3	0.9 (−0.8, 2.5)			
Vaccinated by 18 months	No	8.6 ± 5.2	0	0.24	–	–
	Yes	8.6 ± 4.3	1.0 (−0.7, 2.8)			
CAS baseline	–	–	–		0.7 (0.5, 0.9)	<0.001
SAPAS baseline	<4	–	0	0.77	–	–
	≥ 4	–	−0.2 (−1.3, 1.0)			
GAD-7 baseline (**)	–	–	0.1 (−0.7, 0.9)	0.80	–	–
GAD-7 reduction (**)	–	–	1.3 (0.8, 1.7)	<0.001	(***)	–
sHAI baseline (*)	–	–	−0.2 (−0.6, 0.2)	0.35		
sHAI reduction (*)	–	–	1.1 (0.7, 1.4)	<0.001	0.8 (0.4, 1.2)	<0.001
PHQ-9 baseline (**)	–	–	0.2 (−0.4, 0.7)	0.54	–	–
PHQ-9 reduction (**)	–	–	1.1 (0.7, 1.4)	<0.001	0.8 (0.4, 1.2)	<0.001
OCI-R baseline (*)	–	–	−0.2 (−0.6, 0.2)	0.27	–	–
AUDIT-C baseline	–	–	0 (−0.2, 0.2)	0.94	–	–

CAS, COVID Anxiety Scale; GAD-7, General Anxiety Disorder Assessment 7-item; PHQ-9, Patient Health Questionnaire 9-item; OCI-R, Obsessive–Compulsive Inventory Revised; sHAI, Health Anxiety Inventory Short Form; AUDIT-C, Alcohol Use Disorders Identification Test of Consumption; SAPAS, Standardised Assessment of Personality Abbreviated Scale. Univariate analyses adjust for baseline CAS score.

a Clusters were established using latent profile analysis.^3^

(*) Regression coefficients reported for a 10-unit change in variable.

(**) Regression coefficients reported for a 5-unit change in variable.

(***) GAD-7 reductions were no longer associated with reducing CAS scores in multivariable analysis, and were thus not included in the final model.

Those 61 people who no longer reported symptoms of COVID anxiety also scored lower than individuals who continued to experience symptoms of COVID anxiety on their measures of generalised and health anxiety, depressive symptoms and social dysfunction. There was a gradient between the level of severity of COVID anxiety and co-occurring mental health symptoms (Table 4).

**Table 4:** Comparisons of co-occurring psychopathology scores by severity level of COVID anxiety at 18 months, using analysis of variance significance testing

Psychopathology	No COVID anxiety symptoms (i.e. CAS 0 [s.d.] *n* = 61)	Subclinical COVID anxiety symptoms (i.e. CAS >0, <5 [s.d.] *n* = 80)	Clinically important COVID anxiety symptoms (i.e. CAS >5, <9 [s.d.] *n* = 32)	Severe COVID anxiety symptoms (i.e. CAS >9 [s.d] *n* = 26)	Significance (*P*)
Generalised anxiety (GAD-7)	8.49 [6.26]	9.68 [5.85]	12.84 [4.77]	15.15 [5.03]	<0.001
Depressive symptoms (PHQ-9)	9.64 [7.28]	10.65 [6.81]	14.22 [6.73]	16.54 [5.12]	<0.001
Health anxiety (sHAI)	15.26 [8.38]	17.58 [7.27]	22.0 [6.57]	22.5 [8.77]	<0.001
Alcohol use (AUDIT-C)	3.13 [3.0]	1.96 [2.52]	1.31 [2.0]	2.38 [2.67]	0.009
Social dysfunction (WSAS)	6.10 [8.37]	11.53 [7.70]	17.91 [8.4]	21.0 [7.87]	<0.001

CAS, COVID Anxiety Scale; GAD-7, GAD-7, General Anxiety Disorder Assessment 7-item; PHQ-9, Patient Health Questionnaire 9-item; sHAI, Short Health Anxiety Inventory; AUDIT-C, Alcohol Use Disorders Identification test for Consumption; WSAS, Work and Social Adjustment Scale.

Of 21 people allocated to the CBT-HA treatment arm, 18 (85.7%) provided follow-up responses at the 18-month time point, as did 14 of 19 (73.7%) randomised to the control arm. This feasibility trial was not designed to be powered to detect a difference between groups in outcome measures, because we assessed changes between baseline and 18-month follow-up scores within groups. Akin to the cohort at large, we observed reductions in all measures of poor mental health at 3 and 6 months sustained at 18 months from baseline. In the active arm, 5 of 18 (27.8%) participants continued to report symptoms of COVID anxiety consistent with an impact on daily life (CAS score >5) 18 months later, compared with 5 of 14 (35.7%) in the control arm. There were significant decreases in co-occurring measures of social dysfunction and generalised and health anxiety symptoms in both groups, but significant improvements in depression scale scores in the intervention arm only. For both groups there were significant reductions in the intensity of hand-washing, disinfecting items and COVID media consumption. There were no SAEs recorded from either group (Table 5).

**Table 5:** Outcomes and safety behaviours by intervention arm

Measure	Allocation arm	Baseline mean (s.d.)	3 months (s.d.)	6 months (s.d.)	18 months (s.d.)	Mean difference [95% CI] baseline −18 months
COVID anxiety (CAS)	Intervention	12.0 (2.64)	7.4 (4.50)	5.1 (4.83)	2.8 (3.81)	−9.2 [−7.10, −11.30]
	Control	12.3 (2.71)	8.9 (4.39)	6.4 (4.76)	3.2 (3.38)	−9.1 [−6.94, −11.26]
Depression (PHQ-9)	Intervention	17.05 (3.69)	9.59 (5.97)	9.20 (4.98)	8.23 (6.28)	−8.82 [−5.53, −12.10]
	Control	14.26 (5.04)	12.67 (4.94)	12.94 (8.05)	10.71 (7.04)	−3.55 [0.47, −7.57]
Generalised anxiety (GAD-7)	Intervention	16.57 (3.14)	11.53 (5.60)	9.55 (4.95)	9.72 (6.03)	−6.85 [−3.79, −9.90]
	Control	15.84 (3.80)	13.53 (5.80)	12.44 (7.35)	10.79 (6.92)	−5.05 [−1.22, −8.88]
Health anxiety (sHAI)	Intervention	27.33 (4.26)	19.94 (6.76)	16.50 (6.00)	18.78 (7.03)	−8.55 [−4.84, −12.23]
	Control	27.63 (5.60)	26.67 (6.57)	23.10 (7.81)	21.93 (8.43)	−5.70 [−0.72, −10.68]
Social dysfunction (WSAS)	Intervention	20.43 (5.63)	14.0 (6.53)	10.70 (6.73)	9.33 (8.94)	−11.10 [−6.32, −15.88]
	Control	21.95 (4.31)	20.20 (7.53)	20.94 (10.67)	13.86 (10.11)	−8.09 [−2.82, −13.35]
Not leaving the home (0−4)	Intervention	2.71 (0.56)	2.60 (0.62)	2.25 (0.72)	2.28 (0.75)	−0.44 [−0.86, −0.01]
	Control	2.89 (0.66)	2.59 (0.83)	2.88 (0.62)	2.29 (0.91)	−0.61 [−1.17, −0.052]
Watching COVID news (0−4)	Intervention	3.52 (1.1)	2.58 (1.18)	2.30 (.80)	1.72 (0.67)	−1.80 [−2.40, −1.21]
	Control	3.89 (0.88)	3.40 (0.83)	3.0 (0.97)	2.57 (0.85)	−1.32 [−1.95, −0.70]
Disinfecting items (0−4)	Intervention	2.90 (1.0)	2.24 (1.25)	1.70 (1.13)	1.67 (1.28)	−1.24 [−1.99, −0.48]
	Control	3.05 (1.10)	2.60 (1.12)	2.43 (1.15)	1.86 (0.95)	−1.12 [−1.93, −0.46]
Washing hands (0−4)	Intervention	3.24 (0.54)	2.76 (0.66)	2.34 (0.59)	2.33 (0.84)	−0.91 [−1.36, −0.45]
	Control	3.16 (0.50)	2.87 (0.64)	2.75 (0.68)	2.64 (0.63)	−0.52 [−0.92, −0.11]

CAS, COVID Anxiety Scale; GAD-7, General Anxiety Disorder Assessment 7-item; PHQ-9, Patient Health Questionnaire 9-item; sHAI, Short Health Anxiety Inventory; AUDIT-C, Alcohol Use Disorders Identification test for Consumption; WSAS, Work and Social Adjustment Scale.

**Fig. 2:**
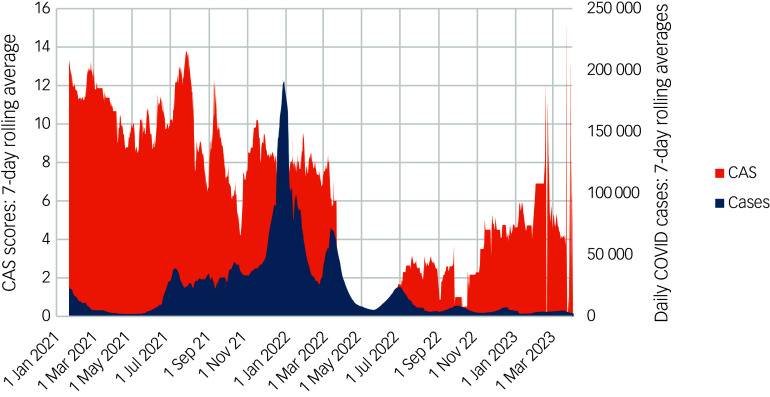
All participant COVID Anxiety Scale (CAS) responses (*n* = 884) by date compared with 7-day rolling average daily cases. England and Wales case rates only, because Scotland and Northern Ireland stopped reporting daily cases in May 2022.

In *ad hoc* univariate analyses, the number of 7-day rolling COVID-19 cases (coefficient 0, *P* < 0.0001), but not hospitalisations, was positively associated with severity scores of COVID anxiety. For daily case rates, the addition of an ordinal time point variable (representing follow-up collection time point: 3, 6 or 18 months) to the model tempered the association of daily case rates with COVID anxiety severity to non-significance (coefficient 0, *P* = 0.41) (Fig. 2).

## Discussion

This study has shown that, among a sample of people with severe COVID anxiety, there were meaningful improvements in COVID anxiety symptoms over time and, for around 1 in 3, a complete resolution 18 months later. There were corresponding declines in co-occurring mental health symptoms and in the unhelpful forms of COVID protective behaviours. However, for almost 4 in 10 there remained a degree of COVID anxiety that had an impact on daily life, and more than 1 in 10 continued to experience a severe degree of symptoms 18 months later. This was the situation, at a time more than 1 year after the ending of all social restrictions and public health enforcement in the UK.

### COVID anxiety, mental health and daily functioning

Despite significant improvements in co-occurring mental health scores over time, this population’s 18-month PHQ-9 and GAD-7 mean scores were still more than double the nationally representative figures over this period.^27^ Indeed the 61 people in this cohort who no longer reported any symptoms of COVID anxiety also continued to report co-occurring mental health symptoms on average to a greater degree than the general population.^27^ This might suggest that people who at one time experienced severe levels of COVID anxiety may also be a population with longer-standing mental health difficulties, or who developed persistent mental health difficulties after their severe COVID anxiety improved. Despite much being written on COVID and mental health, there is little robust evidence to support either of these positions. Studies exploring the associations between COVID anxiety and symptoms of other mental health disorders appear to show a complex connection, probably bidirectional between COVID anxiety and other anxiety states.^10,28^ In some longitudinal studies, COVID-related fear at the beginning of the pandemic was predictive of subsequent poor mental health,^29^ but the evidence which established that mental health disorders were predictive of COVID anxiety is seemingly limited.^30^ There is much more evidence from these studies that existing mental health disorders predicted poorer mental health and stress during the pandemic, although this is rarely demonstrated specifically to be COVID anxiety.

The cohort average level of impairment in socio-occupational functioning (as reported by WSAS) also remained in the ‘moderate impairment’ range at 18 month follow-up, and of particular concern were the 11% of parents in this cohort who continued not to send their children to school, despite being required to do so, due to fears around COVID-19. In the UK, school attendance declined notably after the pandemic and has not yet returned to pre-pandemic levels.^31^ The present study provides some evidence therefore that, in a small number of cases, parental fear of COVID might continue partly to explain this phenomenon.

### Predictors of the trajectory of severe COVID anxiety

This is the first study to have examined the trajectory and factors associated with changes in COVID anxiety over a long follow-up period among people with the most severe levels of COVID anxiety, and corroborates other studies’ findings that COVID-related anxiety improves progressively over time.^15–18^ Previous studies have identified a host of factors that appear to be associated with the severity of COVID anxiety during the pandemic, including older age, non-White ethnicity, personality traits, female gender and general levels of both physical and mental health.^15,16^ Our study builds on this by suggesting that increasing age and being from a heightened-risk ethnic minority background also appear to prolong the severity of COVID anxiety. It is possible that perceived risk accounts for these observations, but we note that other factors contributing to individuals’ objective COVID-19 risk profile, including having an at-risk health condition and vaccination status, do not appear to affect the trajectory of severe COVID anxiety in this study. Furthermore, the level of perceived threat in some studies has been shown to be associated with levels of COVID anxiety,^32^ and for some researchers it therefore stood to reason that greater numbers of reported daily cases, hospitalisations or deaths might create the impression of greater threat for some people, and therefore high COVID anxiety. Indeed, cross-sectional studies from a number of countries, including the UK, showed correlation between daily COVID cases and levels of depressive, generalised anxiety and trauma-related symptoms.^33^ Contrary to this trend, we did not identify an association between 7-day rolling COVID-19 cases or hospitalisations and COVID anxiety in the UK. This complements the one other longitudinal study examining the association of COVID-19-associated daily death rate and COVID anxiety, where there was no association observed.^18^

We previously conducted analysis on this same cohort at the 6-month time point,^9^ which identified that older age, higher baseline levels of generalised and health anxiety and greater reductions in health anxiety and depressive symptoms were significantly associated in multivariable models with greater reductions in COVID anxiety. These findings were again demonstrated 1 year later at the 18-month follow-up time point; however, the effect of baseline mental health scores no longer became predictive of the longer-term trajectory of severe COVID anxiety. Taken together, we suggest that older adults and people from minority ethnic backgrounds at heightened risk ought be accorded special consideration for any targeted support, given that these demographics appear to be associated with both increased severity of COVID anxiety and its persistence.

#### The effect of psychological interventions on the trajectory of severe COVID anxiety

There is some evidence from this cohort to signal that, in the long term, COVID anxiety reduces notably over time for most people irrespective of a CBT intervention (although co-occurring mental health measures and COVID-associated safety behaviours may change differentially). Other CBT interventions have demonstrated small but significant benefits towards COVID anxiety at short follow-up periods.^19,21^ We suspect that, in the long term, any potential benefit of a psychological intervention on levels of COVID anxiety may be overshadowed by the greater benefit of time. However, this does not mean that short-term improvements in COVID anxiety, or improvement in co-occurring psychopathology by other means, are not important exercises for reducing distress and improving the functioning and quality of life among people with severe pandemic anxiety.

#### Habituation, and implications for the treatment of severe COVID anxiety

Unlike other recent pandemics that have been studied for their effects on mental health and in formulating pandemic anxiety, principally H1N1 (swine flu), the SARS-CoV-2 coronavirus became endemic. The stimulus for COVID anxiety has not disappeared. Given the findings of this and other studies, that COVID anxiety appears to improve in time despite the maintained presence of stimuli, we agree with other authors that evidence supports a habituation process being at play for most people with COVID anxiety.^18^ Indeed, there is some evidence from care workers of reduced post-traumatic responses after repeated outbreaks,^34^ and in Germany COVID news consumption followed case rates in-step early in the pandemic but became increasingly disassociated as time went on, demonstrating, as those authors suggest, a habituation effect.^35^ In line with this, we have shown that symptoms of COVID anxiety and COVID-19-specific safety behaviours both steadily decline over time, despite variable rates of national COVID cases during this time.

A ‘regression to the mean’ explanation has been postulated by some authors to explain these repeated findings of improving mental health scores.^12^ Naturally we are unable to demonstrate a causal association as to whether reducing safety behaviours contributed to diminished COVID anxiety symptoms or vice versa. Nonetheless the process of habituation, towards response extinction, necessitates a reduction in safety behaviours^36^ and, notably with COVID anxiety, the behaviour of self-isolation, while potentially effective, when followed so strictly and out of keeping with public health guidance is a perpetuating factor of anxiety.^37^ There is evidence that lockdown periods were associated with greater levels of distress in part for this reason.^38^

Trial interventions supporting people with severe COVID anxiety have been attempted with a handful of psychological approaches, but many other therapeutic opportunities, such as improving a sense of being socially supported, went untested. Mechanistic therapeutic studies are lacking. Supporting the habituation to extinction process by improving distress tolerance,^39^ reducing propagative and ineffective safety behaviours and graded exposure out of self-isolation may therefore be appropriate targets in the treatment of pandemic anxiety.^32,37^

Given our findings that greater decreases in depressive and health anxiety symptoms were associated with greater declines in COVID anxiety, we might suggest that there is cause to believe that established mental health interventions proven to reduce these symptoms have a theoretical basis for also improving persistent COVID anxiety. In addition, of note reciprocally, randomised clinical trials of interventions specifically targeting COVID anxiety, particularly online CBT-based interventions,^9,19,21^ appear to improve other mental health outcomes too.

### Limitations

This study is limited in several ways. First, given our recruitment procedure, the generalisability of the study may be limited: using mainly online and text message-based advertisements and an online platform may have posed a barrier to certain groups, perhaps older adults and those without access to the internet, in hearing of the study and engaging with it. Second, we could not account for changes in participants’ mental health prior to the start of the pandemic, nor the trajectory their mental health may have been on before recruitment. Third, some factors likely to partially explain our findings went uncollected. For example, we did not collect data on levels of social support and connectedness, which appeared to play a role in predicting the severity of COVID anxiety in other studies.^40^ Finally, while our nested trial was able to demonstrate the feasibility of the intervention, it was not powered *ex ante* to detect changes in clinical or behavioural outcomes.

### Future directions

In conclusion, almost 40% of people with debilitating thoughts and feelings about COVID-19 in this sample during the British third wave of the pandemic continued to experience levels of COVID anxiety associated with social and occupational dysfunction 18 months later and, for 13%, this remained severe. Older adults and people from minority ethnic backgrounds that conferred greater risk from COVID-19 were significantly slower to recover, and ought to be considered for targeted support. Reducing co-occurring health anxiety and depressive symptoms with established interventions might be a target for supporting adults with pandemic-related anxiety in the future.

## Data Availability

Data relating to the present analysis are available from the corresponding author on reasonable request.
